# Assessment of computer-controlled local anesthetic delivery system for pain control during restorative procedures: A randomized controlled trial

**DOI:** 10.15171/joddd.2019.045

**Published:** 2019

**Authors:** Hrishikesh Saoji, Mohan Thomas Nainan, Naveen Nanjappa, Mahesh Ravindra Khairnar, Meeta Hishikar, Vivek Jadhav

**Affiliations:** ^1^Department of Conservative Dentistry and Endodontics, Bharati Vidyapeeth Dental College & Hospital, Kharghar, Navi Mumbai, Maharashtra, India; ^2^Department of Conservative Dentistry and Endodontics, Vydehi Institute of Dental Sciences, Bangalore, Karnataka, India; ^3^Department of Public Health Dentistry, Bharati Vidyapeeth (Deemed to be University) Dental College & Hospital, Sangli, Maharashtra, India; ^4^DY Patil Dental College, Nerul, Navi Mumbai, Maharashtra, India; ^5^Department of Prosthodontics, CSMSS Dental College and Hospital, Aurangabad, Maharashtra, India

**Keywords:** Anesthesia recovery period, local anesthesia, pain, syringes

## Abstract

***Background.*** . Local anesthesia is given to decrease pain perception during dental treatments, but it may itself be a reason for pain and aggravate the dental fear. Computer-controlled local anesthetic delivery system (CCLADS) is one of the alternatives for decreasing the patients’ pain during local anesthesia. This study compared the time required for the recovery from anesthesia, pain/discomfort during injection and pain/discomfort 24 hours after administering local anesthesia with CCLADS, a standard self-aspirating syringe and a conventional disposable 2-mL syringe.

***Methods.*** The study was conducted on 90 subjects (an age group of 20-40 years), who suffered from sensitivity during cavity preparation. They were randomly divided into three groups of 30 individuals each to receive intraligamentary anesthesia (2% lignocaine with 1:80,000 adrenaline) using either of the three techniques: CCLADS, a standard self-aspirating syringe, or a conventional disposable 2-mL syringe. The onset of anesthesia, time required for recovery from anesthesia (in minutes), pain/discomfort during injection and pain/discomfort 24 hours after administering local anesthesia were recorded.

***Results.*** The time required for the onset of anesthesia and recovery from anesthesia was shorter with CCLADS (4.83±2.31 and 34.2±1.895, respectively) as compared to the standard self-aspirating group (10.83±1.90 and 43.5±7.581, respectively) and the conventional group (11.00±2.03 and 43.5±6.453, respectively) (P<0.001). The patients in the CCLADS group experienced no pain during local anesthesia administration as compared to the patients in the self-aspirating and conventional groups. The CCLADS and self-aspirating groups showed lower pain response as compared to the conventional group for pain after 24 hours.

***Conclusion.*** CCLADS can be an effective and pain-free alternative to conventional local anesthetic procedures.

## Introduction

Pain is a multidimensional entity influenced by psychological and physiological factors. Effective pain control during dental treatment is vital as it determines the behavior of the patient for the rest of the appointment.^1^ Pain-related behavior may either increase in the successive appointments or an adjustment to the painful stimulus may occur. The most routinely employed method to avoid pain during dental treatment is the administration of a local anesthetic agent. Local anesthesia can be administered by various techniques, such as local infiltration, nerve block, intraligamentary injection, etc.

However, the administration of anesthetic agents by these techniques itself is not 100% pain-free. Adjunctive topical gel/spray application, use of thinner needles, cartridge syringe injections, jet injections and CCLADS are some of the methods employed to minimize this discomfort.^2^ CCLADS is a device that can inject local anesthetic agents into the tissues at a set speed. The primary benefits of these CCLADS devices can be attributed to the ability of administering a small amount of the local anesthetic solution with a stable infusion mode, thus minimizing the discomfort associated with less controlled injections. The CCLADS devices are well tolerated by the patients, produce less disruptive behavior, and have been shown to be successfully used for restorations, pulpal therapies and extractions in adult and pediatric dentistry.^3,4^ Available literature shows the efficacy of reducing pain experience while using CCLADS for administering local anesthesia across the globe and in India.^2,5,6^ However, very few studies have evaluated the time required for recovery from anesthesia and postoperative pain response after administering local anesthesia using CCLADS.^2^

Hence, the present study was designed to evaluate and compare the time required for the onset of anesthesia, the time required for recovery from anesthesia, pain/discomfort during injection and pain/discomfort 24 hours after administering local anesthesia with CCLADS, a standard self-aspirating syringe and a conventional disposable 2-mL syringe.

## Methods

### Study design and study setting (Figure 1) 

**Figure 1:**
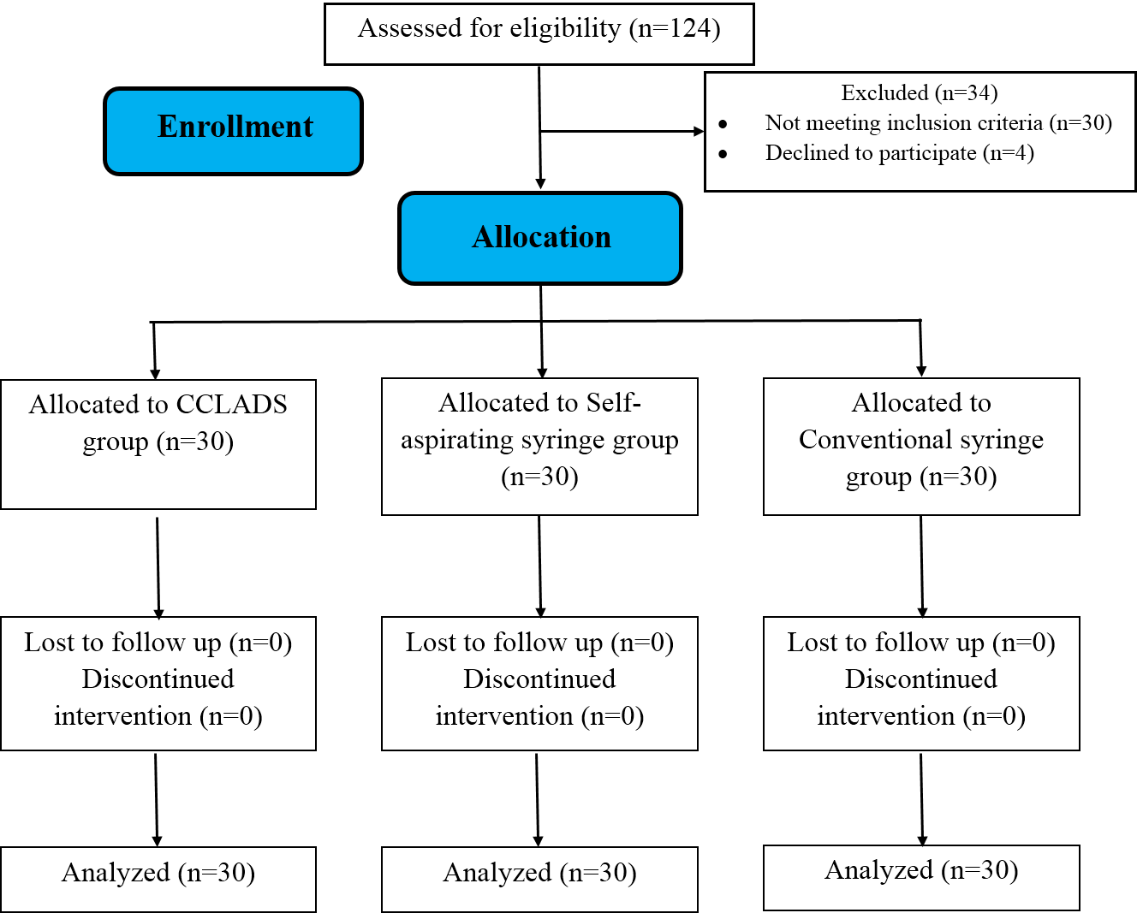
CONSORT 2010 flowchart of the study.

**Figure 2:**
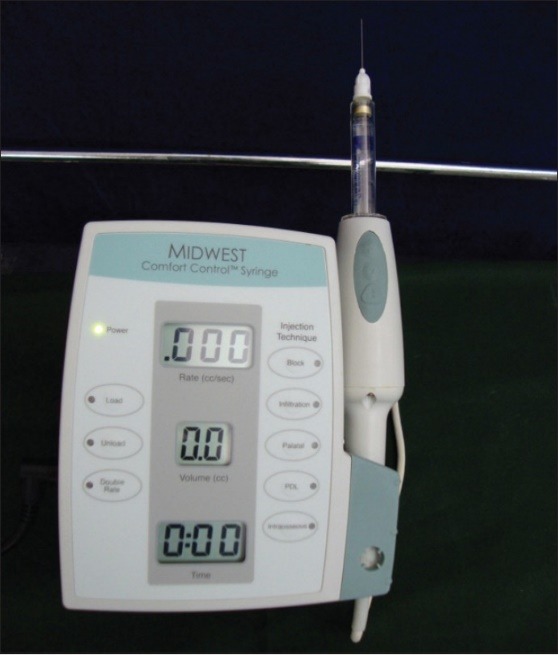
Comfort Control Syringe.

**Figure 3:**
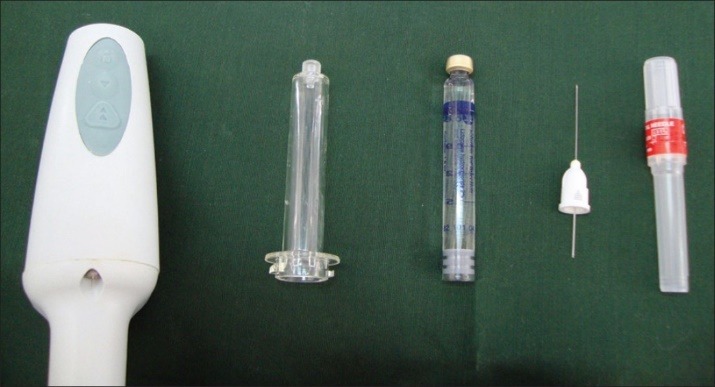
Parts of Comfort Control Syringe Handpiece.

This randomized controlled trial was carried out on 90 subjects in the Department of Conservative Dentistry and Endodontics. The study protocol was designed and implemented considering the Declaration of Helsinki – ethical principles for medical research involving human subjects (adopted by the 18^th^ General Assembly of World Medical Association [WMA], Helsinki, Finland, June 1964 and last amended by the 64^th^ WMA General Assembly, Fortaleza, Brazil, October 2013). The study protocol was approved by the ethics committee of Vydehi Institute of Dental Sciences and Research Center (VIDS/ACM/1177/2011). Written consent was obtained from all the subjects after being informed about all the procedures and possible discomfort. All the participants participated in the study voluntarily and were allowed to exit the study at any time.

### Study population 

The study was conducted on 90 subjects (both males and females in the 20‒40-year age group), visiting the Department of Conservative Dentistry and Endodontics. These 90 subjects were randomly divided in to three equal groups:

1. Group 1: 30 subjects receiving anesthesia with CCLADS [Comfort Control Syringe (MIDWEST), DENTSPLY International, Des Plaines, U.S.A.] with a 27-gauge needle (Figures 2 and 3)
2. Group 2: 30 subjects receiving anesthesia with a standard self-aspirating syringe [SEPTODONT, Saint Fossès Cidex, France] with a 27-gauge needle
3. Group 3: 30 subjects receiving anesthesia with a normal disposable 2-mL syringe [UNOLOK, Faridabad, India] with a 27-gauge needle

### Inclusion criteria

Subjects in need of restorative treatment on vital lower molar teeth, subjects 20‒40 years of age, subjects with periodontally healthy teeth and subjects undergoing complete physical examination in the past 12 months, and subjects with good health without contraindications for local anesthesia were included.

### Exclusion criteria

Subjects with deep caries or caries near pulp, subjects with non-vital teeth, subjects with periodontally compromised teeth, pregnant females, subjects with neurogenic disorders, subjects taking medications that would alter pain perception, and subjects with a history of previous infarction events, a history of percutaneous coronary revascularization within the preceding 6 months were excluded.

### Sample size

The sample size was calculated using GPower software. The required effect size was determined from the data obtained from a previous study conducted by Dulger et al.^7^ Keeping the level of significance at 5% and considering 99% power of the study, the required sample size was estimated at 30 per group.

### Randomization and blinding

Patients who fulfilled the inclusion criteria were randomly assigned to one of the study groups. Randomization was carried out using the lottery technique by a person who was not a part of the study. However, blinding could not be achieved because of the nature of the study. Only, the analyst was kept blind regarding the patient group allocation.

### Intervention and measurements

Clinical evaluations were carried out in each eligible patient before commencement to evaluate whether sensitivity was already present. Patients who were suffering from sensitivity prior to cavity preparation were not included in the study. Patient who reported sensitivity during cavity preparation underwent sensitivity testing procedures. Sensitivity was evaluated using a calibrated pulp tester by a trained examiner who was blinded to the study groups. The subjects were then injected with 2% lignocaine with 1:80,000 adrenaline, using the allotted injection technique; 0.2 mL of the solution per root was deposited into periodontal ligament through the mesiobuccal and distolingual gingival sulcus. The type of injection and the number of subjects who experienced anesthetic success, time for the onset of anesthesia and discomfort or pain were recorded. Thereafter, all the subjects received restorative treatments according to their treatment plan.

A pre-ordered visual analogue scale (from 0 to 10; 0 = no pain/sensitivity; 1‒3 = mild pain; 4‒6 = moderate pain; 7-9 = severe pain; 10 = very severe pain) was used to account for intraligamentary injection causing different degrees of pain during injection. Testing for anesthetic success (no symptoms on electric pulp tester) and the time required for recovery from anesthesia (the first response to electric pulp testing) was evaluated with an electric pulp tester after 1, 5, 10, 15, 20, 25, 30, 35, 40, 45, 50, 55 and 60 minutes, and the presence or lack of sensitivity was recorded, and any additional injection required was recorded.^8^

The subjects were re-called after 24 hours, were interviewed and local examination was carried out to check whether or not the patients had any postoperative discomfort at injection site and pain during chewing. Each participant was again asked to rate their residual discomfort on a visual analogue scale of 0‒10.

### Statistical analysis

The data were compiled and analyzed using SPSS 20. The level of significance was kept at 5%. Frequency distribution was employed to present pain experience among the subjects in each group. The time required for recovery from anesthesia among the three study groups was compared using Kruskal-Wallis test followed by Mann-Whitney test for pairwise comparisons. Pain experience between the study groups was compared using Mann-Whitney test.

## Results

Table 1 shows the comparison of the onset of anesthesia between the three groups. The results showed rapid onset (in minutes) in the CCLADS group (4.83±2.31 minutes), followed by the self-aspirating syringe (10.83±1.90 minutes) and the conventional syringe (11.00±2.03 minutes). This difference in time required for the onset of anesthesia among the three groups was statistically significant (P=0.001). When compared pairwise, the CCLADS showed a significant difference in the onset of anesthesia from both the self-aspirating syringe (P=0.001) and the conventional syringe (P=0.001). However, the difference between the self-aspirating syringe and the conventional syringe was not significant (P=0.572).

**Table 1:** Comparison of the onset of anesthesia (in minutes) between the different systems

**Systems used**	**Mean ± SD**	**Min‒Max**	**P-value**	**Intergroup comparison**
**Self-aspirating syringe (SAS)**	10.83±1.90	10‒15	0.001*	SAS vs. CS P=0.741
**Conventional syringe (CS)**	11.00±2.03	10‒15		SAS vs. CCLADS P=0.001*
**Computer-controlled device (CCLADS)**	4.83±2.31	1‒10		CS vs. CCLADS P=0.001*

Kruskal-Wallis test; Mann-Whitney test; * indicates significant at P≤0.05

Table 2 shows the comparison of the time required for recovery from anesthesia between the three groups. The results showed the fastest recovery response (in minutes) in the CCLADS group (34.2±1.895 minutes), followed by the self-aspirating syringe (43.3±7.581 minutes) and the conventional syringe (43.5±6.453 minutes) groups, respectively. This difference in the time required for recovery from anesthesia among the three groups was statistically significant (P=0.001). When compared pairwise, CCLADS exhibited a significant difference in the time required for recovery from anesthesia from both the self-aspirating syringe (P=0.001) and the conventional syringe (P=0.001). However, the difference between the self-aspirating syringe and conventional syringe was not significant (P=0.572). Five subjects in the self-aspirating syringe group and six subjects in the conventional syringe group required additional injections.

**Table 2:** Comparison of the first anesthetic recovery response (in minutes) between the different systems

**Systems used**	**Mean ± SD**	**Min‒Max**	**P-value**	**Intergroup comparison**
**Self-aspirating syringe (SAS)**	43.3±7.581	40‒60	0.001*	SAS vs. CS P=0.572
**Conventional syringe (CS)**	43.5±6.453	40‒60		SAS vs. CCLADS P=0.001*
**Computer-controlled device (CCLADS)**	34.2±1.895	30‒35		CS vs. CCLADS P=0.001*

Kruskal-Wallis test; Mann-Whitney test; * indicates significant at P≤0.05

Table 3 demonstrates the pain perception during injection between different systems as determined by VAS. All the subjects in the CCLADS group (100%), 90% of the subjects in the self-aspirating syringe group and 80% of the subjects in the conventional syringe group reported no pain during injection. Pairwise comparisons showed no significant differences between the groups tested (P>0.05), except for the CCLADS and conventional systems (P=0.03).

**Table 3:** Comparison of pain experience (in %) during the injection between the different systems as determined by VAS

**Systems used**	**No pain**	**Mild pain**	**Moderate pain**	**Intergroup comparison**
**Self-aspirating syringe (SAS)**	90	10	0	SAS vs. CS P=0.06
**Conventional syringe (CS)**	80	0	20	SAS vs. CCLADS P=0.236
**Computer-controlled device (CCLADS)**	100	0	0	CS vs. CCLADS P=0.03*

Mann-Whitney test; * indicates significant at P≤0.05

Table 4 shows the pain experience after 24 hours of injection between the different systems as determined by VAS. Pain experience was similar for the CCLADS and self-aspirating syringe groups (P=1.000). 90% of the subjects from both groups experienced no pain/discomfort after 24 hours as compared to 63% of the subjects in the conventional group. A significant difference was noted when CCLADS was compared with the conventional syringe (P=0.019), and the self-aspirating syringe was compared with the conventional syringe (P=0.019) for pain after 24 hours as determined by VAS.

**Table 4:** Comparison of pain experience (in %) 24 hours after injection between the different systems as determined by VAS

**Systems used**	**No pain**	**Mild pain**	**Moderate pain**	**Intergroup comparison**
**Self-aspirating syringe (SAS)**	90	10	0	SAS vs. CS P=0.019*
**Conventional syringe (CS)**	63.3	16.7	20	SAS vs. CCLADS P=1.000
**Computer-controlled device (CCLADS)**	90	10	0	CS vs. CCLADS P=0.019*

Mann-Whitney test; * indicates significant at P≤0.05

## Discussion

The literature search shows scarcity of data regarding the time required for recovery from anesthesia and postoperative pain after local anesthetic administration using the CCLAD system. Therefore, the present study was planned to determine the anesthetic efficacy of CCLADS compared with a self-aspirating syringe and a conventional syringe. Conventional dental nerve block technique or local infiltration method exhibits certain drawbacks.^9^ To overcome these drawbacks, alternative methods, like periodontal ligament or intraligamentary injection and intraosseous anesthetic injection technique, were introduced. The periodontal ligament injection (PDL) or intraligamentary injection technique appears to be the most consistently reliable technique in achieving clinically adequate pulpal anesthesia and offers many advantages in comparison to the conventional dental nerve block and infiltration anesthesia methods.^10^ The PDL injection method of anesthetizing an individual tooth is utilized to avoid the undesirable consequences of regional block anesthesia. The use of a PDL injection during restorative dental procedures allows for a quick onset of anesthesia, usually immediately, as well as profound anesthesia for an adequate length of time to perform most routine procedures. In addition, the PDL injection techniques could serve as adjuncts to routine injections to alleviate patient discomfort and pain. Therefore, the present study was designed to assess the efficacy of periodontal ligament injection in patients having sensitivity during cavity preparation for restorative treatments.

The results of the present clinical study revealed that Midwest comfort controlled syringe (CCLADS) was highly effective in achieving anesthesia and pain control measures. The length of time needed to produce an anesthetic effect was not more than 10 minutes in all the patients in the CCALD group, while a longer period was needed when using either a traditional dental syringe or a standard self-aspirating syringe. It is noted that 10 minutes is the clinically accepted period to wait for the effect of anesthesia, while a longer duration is usually considered by both the dentist and the patient as too long. Another important aspect to note was that additional injections were needed in 6 patients in the normal disposable 2-mL syringe group and 5 patients in the standard self-aspirating syringe group, compared to the Midwest comfort control syringe, which required no additional injections. This could be due to the fact CCLADS delivers a precise rate of anesthetic solution, while maintaining constant pressure and time, into the periodontal space throughout the phase of anesthesia.

The results of the present study showed better results for CCLADS in terms of both the times required for the onset of anesthesia and recovery from anesthesia. Subjects in the CCLAD group experienced faster onset of anesthesia (4.83 minutes) and showed significant differences from the other techniques. The rapid onset of anesthesia provides more comfort by reducing stress among patients. These results are in contrast to the studies conducted by Palm et al^11^ and Kandiah et al,^12^ where no significant differences were seen in the onset of anesthesia between the computer-controlled technique and the conventional technique. The differences in the results might be attributed to different techniques used in the administration of local anesthesia (nerve block and infiltration). The subjects in the CCLADS group experienced faster recovery (34.2 minutes) from local anesthesia as compared to the subjects in the self-aspirating syringe group (43.3 minutes) and the subjects in the conventional group (43.5 minutes). Similar results were seen in a study by Beneito-Brotons et al,^13^ where the time required for recovery from anesthesia for injection by computer-controlled system was about 1.6 minutes as compared to the conventional technique (199 minutes). However, a study by Lee et al^14^ showed no significant difference in the time required for recovery from anesthesia between the CCLAD and conventional local anesthesia techniques.^14^

In the present study, subjects in the CCLADS group did not suffer from pain during anesthesia administration, and this pain experience significantly differed from the conventional group (P=0.03). Similar results were seen in a study conducted to assess the levels of anxiety and pain associated with the computer-driven system using VAS.^15^ The available literature shows that the majority of previous studies conducted to compare the computer-controlled injection systems with conventional syringes for pain during injection showed results in favor of computer-controlled injection systems.^2,16,17^ These results were contradicted in a study by Goodell et al,^19^ where the conventional atraumatic syringe injection technique was found to be superior to a controlled injection pressure system in pain perception and procedure tolerance and in reducing post-injection dental anxiety.^18^ The present study did not show any difference between the computer-controlled and self-aspirating syringe in the pain during injection. Previous studies also showed improved pain responses with the use of CCLADS as compared to cartridge syringes. The possible reason stated was the injection pressure with cartridge syringe, which is difficult to control.^16,17^ Saloum et al^19^ reported that injection with self-aspirating syringe was more painful than injection with computer-controlled devices.^19^ The operator technique and tactile skill in syringe injections and site of injection (right or left) were the possible reasons for differences in pain perception in the study by Saloum et al.

Pain differed significantly between the conventional and CCLADS and between the conventional and self-aspirating syringe 24 hours after injection. These findings are contradictory to the study conducted by Najlaa et al,^20^ which showed no significant difference in postoperative complications between CCLADS and conventional systems. No other study has compared the postoperative pain after 24 hours of injection using the CCLAD system with either the self-aspirating syringe or the conventional technique.

It can be noted that similar-gauge needles were used for all the three systems in the present study, whereas different-gauge needle use has been reported in most of the previously conducted studies. The study was performed without blinding the operator and participants, which can be considered as a limitation of the study. An attempt was made to minimize this bias by appointing an independent observer who recorded the time required for recovery from anesthesia and the pain response of the patients. Therefore, the results of the study could not be generalized to the entire treatment procedure.

## Conclusion

Within the limitations of this study, it can be concluded that the computer-controlled local anesthetic delivery system resulted in more predictable and reliable intraligamentary anesthesia than a self-aspirating syringe and a conventional syringe with respect to the time required for recovery from anesthesia and pain perception. Hence, use of CCLADS can be considered as a promising step towards accomplishing a relatively pain-free dental treatment and also in developing a positive attitude towards dental treatment.

## Author Contribution

HS and MTN designed the study. HS, NN and MH performed the literature review. HS and MH performed the experimental procedure. MRK carried out the statistical analyses and interpreted data. MRK and VJ drafted the manuscript. All the authors critically revised the manuscript for intellectual content. All the authors have read and approved the final manuscript.

## Acknowledgments

None.

## Funding

Not applicable.

## Competing Interests

The authors declare no competing interests with regards to the authorship and/or publication of this article.

## Ethics Approval

The study protocol was approved by the ethics committee of Vydehi Institute of Dental Sciences and Research Center (VIDS/ACM/1177/2011).
